# Ancestry and dental development: A geographic and genetic perspective

**DOI:** 10.1002/ajpa.23351

**Published:** 2017-11-15

**Authors:** Brunilda Dhamo, Lea Kragt, Olja Grgic, Strahinja Vucic, Carolina Medina‐Gomez, Fernando Rivadeneira, Vincent W.V. Jaddoe, Eppo B. Wolvius, Edwin M. Ongkosuwito

**Affiliations:** ^1^ The Department of Oral & Maxillofacial Surgery, Special Dental Care and Orthodontics Erasmus University Medical Centre Rotterdam the Netherlands; ^2^ The Generation R Study Group Erasmus University Medical Centre the Netherlands; ^3^ The Department of Internal Medicine Erasmus University Medical Center Rotterdam the Netherlands; ^4^ The Department of Epidemiology Erasmus University Medical Centre the Netherlands

**Keywords:** dental age, genes, geography, maturation, origin, tooth

## Abstract

**Objective:**

In this study, we investigated the influence of ancestry on dental development in the Generation R Study.

**Methods:**

Information on geographic ancestry was available in 3,600 children (1,810 boys and 1,790 girls, mean age 9.81 ± 0.35 years) and information about genetic ancestry was available in 2,786 children (1,387 boys and 1,399 girls, mean age 9.82 ± 0.34 years). Dental development was assessed in all children using the Demirjian method. The associations of geographic ancestry (Cape Verdean, Moroccan, Turkish, Dutch Antillean, Surinamese Creole and Surinamese Hindustani vs Dutch as the reference group) and genetic content of ancestry (European, African or Asian) with dental development was analyzed using linear regression models.

**Results:**

In a geographic perspective of ancestry, Moroccan (β = 0.18; 95% CI: 0.07, 0.28), Turkish (β = 0.22; 95% CI: 0.12, 0.32), Dutch Antillean (β = 0.27; 95% CI: 0.12, 0.41), and Surinamese Creole (β = 0.16; 95% CI: 0.03, 0.30) preceded Dutch children in dental development. Moreover, in a genetic perspective of ancestry, a higher proportion of European ancestry was associated with decelerated dental development (β = −0.32; 95% CI: –.44, –.20). In contrast, a higher proportion of African ancestry (β = 0.29; 95% CI: 0.16, 0.43) and a higher proportion of Asian ancestry (β = 0.28; 95% CI: 0.09, 0.48) were associated with accelerated dental development. When investigating only European children, these effect estimates increased to twice as large in absolute value.

**Conclusion:**

Based on a geographic and genetic perspective, differences in dental development exist in a population of heterogeneous ancestry and should be considered when describing the physiological growth in children.

## 1 INTRODUCTION

Dental development is a progressive and continuous process determined by interactions of genetic, epigenetic, and environmental factors over time (Townsend & Brook, 2008).

In different geographical areas, populations have shown variations in dental development including different morphology of teeth and other dental anomalies (Dhanrajani, 2002; Hanihara & Ishida, 2005; Uthaman, Sequeira, & Jain, 2015). Characteristics in shape, size, and structure of teeth are recognized as indicators of dental differences in populations. For example, Africans have bigger teeth with thicker enamel, whereas Europeans have smaller teeth and a reduction in tooth mass (Harris & Rathbun, 1991; Shah, Boyd, & Vakil, 1978; Vaughan & Harris, 1992). Aside from variations in dental morphology and anomalies, variations in the rate (e.g., accelerations or decelerations) of dental development have been noted across populations. For example, previous work has shown that Africans precede Europeans in the timing of tooth formation (Harris & McKee, 1990; Roberts, 1969), by achieving each of the stages of dental development about 5% earlier (Harris & Rathbun, 1991). Among the studied populations, Australians have the fastest dental development and Koreans have the slowest, a difference that has been attributed to ecological and genetic factors (Chaillet, Nystrom, & Demirjian, 2005). Furthermore, decelerated dental development is recognized in northern populations, whereas accelerated dental development is shown in tropical populations (Roberts, 1978).

Genes are known to play a predominate role on dental development (Townsend & Brook, 2008). However, because of geographical diversity in climate and latitude, physical factors such as temperature, sun exposure, and humidity have shown to associate with variations in growth and also dental development among populations (Baker, 1966; Mazess, 1975; Roberts, 1978; Smithers & Smit, 1997).

Thus, a geographic and genetic approach of ancestry is necessary to explain the variations in timing of dental development. In addition, the recognition of differences in dental development within a population is important to better understand the environmental influence and genetic implications (Garn, Lewis, & Blizzard, 1965; Garn, Lewis, & Kerewsky, 1965; Roberts, 1969; Townsend, Hughes, Luciano, Bockmann, & Brook, 2009).

Beyond the above‐mentioned facts, because of limited data on dental development, the literature provides little evidence about the influence of ancestry on dental development within populations (Liversidge, Speechly, & Hector, 1999; Nystrom, Ranta, Kataja, & Silvola, 1988; Roberts, 1978). Therefore, in a large number of subjects, as part of a multi‐ethnic population‐based prospective cohort study, we aimed to investigate the influence of ancestry on dental development, based on a geographic and genetic perspective.

## 2 MATERIALS AND METHODS

### 2.1 Study design

This study was embedded in the Generation R Study, a multi‐ethnic, population‐based, prospective prenatal cohort which was initiated to identify early environmental and genetic determinants of growth, development, and health (Jaddoe et al., 2012; Kooijman et al., 2016). All children were born between April 2002 and January 2006. Enrollment in the study was aimed at early pregnancy but was allowed until the birth of the child. Data collection in children and their parents included questionnaires, interviews, detailed physical and ultrasound examinations, behavioral observations, magnetic resonance imagining, and biological samples. The Generation R Study has been conducted in accordance with the World Medical Association Declaration of Helsinki and all study phases have been approved by the Medical Ethical Committee of the Erasmus Medical Center, Rotterdam, the Netherlands (MEC‐2012‐165) (Jaddoe et al., 2012).

### 2.2 Study population

In total, 4,447 dental panoramic radiographs (DPRs) taken in 4,447 children at age‐10 assessment, were used to assess dental development. Information about geographic ancestry was available in 3,600 children (1,810 boys and 1,790 girls; mean age 9.81 ± 0.35 years), and information about genetic ancestry was available in 2,786 children (1,387 boys and 1,399 girls; mean age 9.82 ± 0.34 years) (Tables 1a and 1b, Supporting Information Figure S1).

**Table 1:** General characteristics of the study sample

	Geographic ancestries							
	Total (*N* = 3,600)	Dutch (*N* = 2,603)	Cape Verdean (*N* = 132)	*p*‐value	Moroccan (*N* = 232)	*`*‐value	Turkish (*N* = 275)	*p*‐value
Age	9.81 (0.35)	9.78 (0.32)	9.92 (0.48)	*<0.001*	9.90 (0.41)	*<0.001*	9.90 (0.45)	*<0.001*
Sex				0.459		0.123		0.160
Boys	1810 (50.3)	1304 (50.1)	65 (49.2)		126 (54.3)		147 (53.5)	
Girls	1790 (49.7)	1299 (49.9)	67 (50.8)		106 (45.7)		128 (46.5)	
Maternal age	31.04 (4.87)	31.77 (4.46)	29.98 (5.27)	*<0.001*	29.21 (5.13)	*<0.001*	28.30 (5.00)	*<0.001*
Height	141.77 (6.62)	141.98 (6.36)	142.40 (7.91)	0.461	140.14 (6.53)	*<0.001*	140.29 (6.81)	*<0.001*
Weight	35.51 (7.36)	34.66 (6.39)	39.51 (10.33)	*<0.001*	36.59 (8.17)	*<0.001*	38.35 (8.88)	*<0.001*
BMI	17.56 (2.76)	17.11 (2.34)	19.24 (3.48)	*<0.001*	18.51 (3.16)	*<0.001*	19.33 (3.38)	*<0.001*
dmft	0.0 (0.0–6.0)	0.0 (0.0–3.0)	0.0 (0.0–6.0)	*<0.001*	2.0 (0.0–9.0)	*<0.001*	1.5 (0.0–11.0)	*<0.001*
Dental age^a^	10.33 (0.84)	10.25 (0.78)	10.46 (0.93)	*0.003*	10.53 (0.95)	*<0.001*	10.61 (1.03)	*<0.001*
Dental age^b^	11.21 (1.13)	11.10 (1.07)	11.28 (1.11)	*<0.001*	11.46 (1.18)	*<0.001*	11.59 (1.29)	*<0.001*
Dental age^c^	10.59 (0.93)	10.49 (0.86)	10.78 (1.11)	*<0.001*	10.83 (1.03)	*<0.001*	10.95 (1.14)	*<0.001*
Hypodontia	184 (5.1)	137 (5.3)	2 (1.5)	*0.022*	12 (5.2)	0.438	17 (6.2)	0.388
Dental anomalies of position	91 (2.5)	68 (2.6)	5 (3.8)	0.275	2 (0.9)	0.065	4 (1.5)	0.167

	Total (*N* = 3,600)	Dutch (*N* = 2,603)	Dutch Antillean (*N* = 113)	*p*‐value	Surinamese Creole (*N* = 120)	*p*‐value	Surinamese Hindustani (*N* = 125)	*p*‐value
Age	9.81 (0.35)	9.78 (0.32)	9.89 (0.47)	*0.001*	9.85 (0.36)	*0.033*	9.79 (0.31)	0.741
Sex				0.174		0.458		0.237
Boys	1810 (50.3)	1304 (50.1)	51 (45.1)		59 (49.2)		58 (46.4)	
Girls	1790 (49.7)	1299 (49.9)	62 (54.9)		61 (50.8)		67 (53.6)	
Maternal age	31.04 (4.87)	31.77 (4.46)	28.09 (6.36)	*<0.001*	30.83 (5.87)	*0.027*	29.26 (4.63)	*<0.001*
Height	141.77 (6.62)	141.98 (6.36)	142.53 (7.27)	0.370	143.36 (7.52)	*0.021*	140.83 (7.53)	0.052
Weight	35.51 (7.36)	34.66 (6.39)	39.30 (10.50)	*<0.001*	38.19 (8.87)	*<0.001*	34.66 (7.54)	0.996
BMI	17.56 (2.76)	17.11 (2.34)	19.13 (3.68)	*<0.001*	18.41 (3.13)	*<0.001*	17.37 (2.99)	0.226
dmft	0.0 (0.0–6.0)	0.0 (0.0–3.0)	0.0 (0.0–3.0)	0.766	0.0 (0.0–3.6)	0.600	0.0 (0.0–8.9)	*<0.001*
Dental age^a^	10.33 (0.84)	10.25 (0.78)	10.68 (0.98)	*<0.001*	10.54 (0.66)	*<0.001*	10.36 (0.77)	0.130
Dental age^b^	11.21 (1.13)	11.10 (1.07)	11.74 (1.27)	*<0.001*	11.53 (1.00)	*<0.001*	11.28 (1.11)	0.064
Dental age^c^	10.59 (0.93)	10.49 (0.86)	11.02 (1.12)	*<0.001*	10.84 (0.80)	*<0.001*	10.63 (0.90)	0.096
Hypodontia	184 (5.1)	137 (5.3)	4 (3.5)	0.122	6 (5.0)	0.517	6 (4.8)	0.448
Dental anomalies of position	91 (2.5)	68 (2.6)	4 (3.5)	0.352	2 (1.7)	0.396	6 (4.8)	0.121

*Abbreviations*: No = number of participants, dmft = dental caries in deciduous dentition.

Values are percentages for categorical variables, means (SD) for continuous variables with a normal distribution, or medians (95% range) for continuous variables with a skewed distribution; Differences were tested using one way ANOVA and Chi‐square tests for variables with a normal distribution and Kruskal‐Wallis Nonparametric test for variables with a skewed distribution, using Dutch ethnicity as the reference group; Significant *p*‐values are presented in italic font;

a Dental age calculated by the Dutch standard.

b Dental age calculated by the French‐Canadian standard.

c Dental age calculated by the International Demirjian standard.

### 2.3 The assessment of ancestry

The ancestry of children was defined in two ways:

1. *Geographic ancestry*: Information about countries of birth of the parents was obtained by questionnaires. Children of whom both parents were born in the Netherlands were classified as Dutch (*N* = 2,603). The child was of non‐Dutch origin if one or both of the parents were born abroad. If the parents were born in different countries, the country of birth of the mother determined the geographic ancestral background (Netherlands, 2003). This approach has been previously described in detail (Jaddoe et al., 2012). We defined the following non‐Dutch groups: Cape Verdean (*N* = 132), Moroccan (*N* = 232), Turkish (*N* = 275), Dutch Antillean (*N* = 113), and Surinamese (*N* = 245). The Surinamese population consists of persons who originate from Africa (Creoles) and India (Hindustani), therefore we further classified children with a Surinamese geographic ancestry as: Surinamese‐Creole (*N* = 120) or Surinamese‐Hindustani (*N* = 125) based on the origin of the Surinamese parent (Troe et al., 2007).
2. *Genetic ancestry*: Blood samples of the children were collected from the umbilical cord at birth. Where an umbilical cord blood sample could not be collected at birth, a blood sample was obtained by venipuncture during the child's visit to the research center at age‐6 assessment (Kooijman et al., 2016). Genotyping was performed in the Genetic Laboratory of the Erasmus Medical Center, Department of Internal Medicine, Rotterdam, the Netherlands using Illumina HumanHap 610 or 660 Quad chips depending on collection time following manufacturer protocols, and intensities were obtained from the BeadArray Reader (Medina‐Gomez, Felix et al., 2015b). Genetic ancestry was identified by admixture analysis applied in participants of the Generation R Study (Medina‐Gomez, Chesi et al., 2015). This method models the probability of observed genotypes using ancestry proportions and ancestral population allele frequencies. The clustering method was set to group individuals in three ancestral populations (K = 3), corresponding to the expected main Sub‐Saharan African, European, and East Asian ancestry components (International HapMap C, 2003, 2005). Children were assigned to one of the three ancestry groups, labeled after the HapMap Phase II populations, based on their highest fraction of estimated ancestry (i.e., 40.50) proportions. We defined 2,473 children of European origin, 204 children of African origin, and 109 children of Asian origin. Cases that did not reach any significant proportion of the three ancestral populations, were excluded from further analysis (*N* = 48).

### 2.4 Dental development

Dental development was defined using the Demirjian method (Demirjian, Goldstein, & Tanner, 1973). One experienced examiner (B.D) determined the eight stages of development (1 to 8) for each of the seven permanent teeth located in the lower left quadrant excluding the third molar. In the event any permanent tooth in the left mandible was congenitally missing, the stage of development was assessed from the corresponding tooth in the right mandible; if the corresponding right tooth was missing as well, regression equations which take into account the age and sex of the child, and development of the remaining teeth in the lower left quadrant, were applied to assess the stage of development for the missing tooth (Nyström, Aine, Peck, Haavikko, & Kataja, 2000). The obtained stages of development were weighted using three different dental age standards (Dutch standard, French‐Canadian standard, and International Demrijian standard) and subsequently for each standard separately summed to calculate the gender specific maturity scores (Chaillet et al., 2005; Demirjian et al., 1973; Leurs, Wattel, Aartman, Etty, & Prahl‐Andersen, 2005). Finally, standard tables were used to convert the dental maturity scores into dental ages. Dental age calculated by the Dutch standard consistently presented the best approximation with chronological age in our study population, hence it was used as a proxy of dental development in the subsequent statistical analysis.

### 2.5 Covariates

Chronological age of a child was calculated as the interval between the date when the DPR was taken and the date of birth. Information on child's sex and day of birth were available from medical records and hospital registries. As sex is taken in consideration when dental age is calculated, we used sex as a potential confounder only to study the influence of ancestry on the developmental stages of each left mandibular tooth. Hypodontia was ascertained from the DPRs. Children were classified with hypodontia if no sign of tooth formation or calcification was shown in DPR. Most of children who revealed hypodontia had 1–2 absent teeth. Hence, they were not excluded from the study population as Demirjian method takes into account missing teeth. Weight was measured using a mechanical personal scale (SECA, Almere, the Netherlands). Child height was determined in standing position to the nearest millimeter without shoes by a Harpendenstadiometer (Holtain Limited, Dyfed, UK). BMI (kg/m^2^) was calculated using the weight and height measured during the age‐10 assessment. The decayed, missing, and filled teeth index (dmft) was used to assess dental caries when children were 6 years old, a high‐risk age for dental caries in deciduous dentition. The dmft‐score of each child was obtained from intraoral photographs (Elfrink, Veerkamp, Aartman, Moll, & Ten Cate, 2009). Covariates were included in the regression models based on previous literature or a change of >10% in effect estimates.

### 2.6 Statistical analysis

We used the Intra‐Class Correlation (ICC) to test the agreement between two independent examiners, who assessed stages of development (1 to 8) for each of the seven left mandibular teeth in a random subsample of 100 DPRs from the study population. The ICC for the scored teeth ranged between 0.65 and 0.80 which is considered to be a substantial agreement according to the conventional criteria (Landis & Koch, 1977). First incisors were not taken into account because of the absence of variation in the stages of tooth development fitting with the age of the children.

The association between geographic ancestry and dental development (dental age calculated by the Dutch standard) was analyzed using two generalized linear models. In Model 1, we adjusted the association for chronological age. In Model 2, we additionally adjusted for hypodontia, BMI, height, and dmft. This analysis was performed for Cape Verdean, Moroccan, Turkish, Dutch Antillean, Surinamese Creole, and Surinamese Hindustani children with Dutch children as the reference group. The association of each content of genetic ancestry (European, African, Asian) with dental age was analyzed using two multivariate linear regression models adjusted for the same potential confounders. This analysis was performed both in the complete study sample and also in European children only for specificity, as they represented the majority (88.8%) of our study sample.

The association between genetic ancestry and development of each mandibular tooth in the left lower quadrant (the reference quadrant) was analyzed using two ordinal regression models. In Model 1 we adjusted the association for chronological age and sex. In Model 2, we additionally adjusted for hypodontia, BMI, height, and dmft. This analysis was performed for African and Asian children with European children as the reference group.

We tested for interactions of sex and hypodontia with geographic and genetic ancestry in relation to dental age. Since no significant interaction terms were found, we did not stratify our analysis. To check for selection bias, we performed nonresponse analysis (using the one‐way‐Analysis of Variance (ANOVA), Chi‐square test, and Kruskal‐Wallis nonparametric test, depending on the distribution of the data) to test the differences between subjects that were included and subjects that were eligible to be included but were left out because of lack of available data on dental development. The Markov Chain Monte Carlo imputation method (Sterne et al., 2009) was used to reduce potential bias associated with missing data on dmft at the age‐6 assessment in 1,106 children (25%). Five imputed datasets were generated and pooled effect estimates are presented (β; 95% CI). All results were considered statistically significant for a *p*‐value ≤0.05. All statistical analyses in this study were performed using Statistical Package for Social Sciences version 21.0 (SPSS, Chicago, IL, USA).

## 3 RESULTS

### 3.1 General characteristics

#### 3.1.1 Geographic ancestry

Hypodontia was more frequent in Cape Verdean children than in Dutch children (*p* = 0.022). Cape Verdean, Moroccan, Turkish, Dutch Antillean, and Surinamese Creole children had a higher BMI compared to Dutch children (*p* < 0.001). Moroccan and Turkish children were shorter than the reference group (*p* < 0.001), while Surinamese Creole children were taller than the reference group (*p* < 0.001). The dmft was higher in Cape Verdean, Moroccan, Turkish, and Surinamese‐Hindustani children compared with Dutch children (*p* < 0.001) (Table 1a).

The dental age calculated by the Dutch standard was higher in children of Cape Verdean (mean:10.46 years), Moroccan (mean:10.53years), Turkish (mean: 10.61 years), Dutch Antillean (mean: 10.68 years), Surinamese Creole (mean: 10.54 years) descent compared to Dutch children (mean: 10.25 years). In contrast, there was no statistically significant difference in dental age between Surinamese Hindustani children (mean: 10.36 years) and Dutch children (mean: 10.25years).

#### 3.1.2 Genetic ancestry

When compared to children of European ancestry, no significant difference in the frequency of hypodontia was present in children of African (*p* = 0.143) and Asian ancestry (*p* = 0.072). BMI was higher in children of African ancestry compared with children of European ancestry (*p* < 0.001). African children were taller than European children (*p* = 0.001), while Asian children were shorter than European children (*p* < 0.001). The dmft was higher in children of Asian ancestry compared to children of European ancestry (*p* = 0.013) (Table 1b).

**Table 2:** General characteristics of the study sample

	Genetic ancestries					
	Total (*N* = 2786)	Europeans (*N* = 2473)	Africans (*N* = 204)	*p*‐value	Asians (*N* = 109)	*p*‐value
Age	9.82 (0.34)	9.81 (0.34)	9.92 (0.49)	*<0.001*	9.82 (0.32)	0.794
Sex				0.251		0.086
Boys	1387 (49.8)	1243 (50.3)	97 (47.5)		47 (43.1)	
Girls	1399 (50.2)	1230 (49.7)	107 (52.5)		62 (56.9)	
Maternal age	30.91 (4.81)	31.23 (4.58)	28.06 (6.16)	*<0.001*	28.94 (4.68)	*<0.001*
Height	141.87 (6.75)	141.85 (6.60)	143.45 (7.42)	*0.001*	139.30 (7.92)	*<0.001*
Weight	35.47 (7.17)	35.22 (6.83)	39.22 (9.25)	*<0.001*	33.97 (8.28)	0.063
BMI	17.52 (2.66)	17.41 (2.54)	18.90 (3.33)	*<0.001*	17.32 (3.04)	0.713
dmft	0.0 (0.0–7.0)	0.0 (0.0–7.0)	0.0 (0.0–6.8)	0.958	0.0 (0.0–9.6)	*0.013*
European content of ancestry	1.0 (0.1–1.0)	1.0 (0.5–1.0)	0.3 (0.1–0.5)	*<0.001*	0.4 (0.0–0.5)	*<0.001*
African content of ancestry	0.0 (0.0–0.8)	0.0 (0.1–0.4)	0.7 (0.5–1.0)	*<0.001*	0.0 (0.0–0.4)	*<0.001*
Asian content of ancestry	0.0 (0.0–0.5)	0.0 (0.0–0.3)	0.0 (0.0–0.2)	0.132	0.6 (0.5–1.0)	*<0.001*
Dental age^a^	10.34 (0.82)	10.32 (0.82)	10.65 (0.87)	*<0.001*	10.31 (0.77)	0.900
Dental age^b^	11.23 (1.12)	11.19 (1.11)	11.70 (1.19)	*<0.001*	11.21 (1.11)	0.877
Dental age^c^	10.61 (0.92)	10.58 (0.90)	10.98 (1.06)	*<0.001*	10.57 (0.90)	0.922
Hypodontia	149 (5.3)	134 (5.4)	7 (3.4)	0.143	8 (7.3)	0.072
Dental anomalies of position	77 (2.8)	64 (2.6)	7 (3.4)	0.295	6 (5.5)	0.112

*Abbreviations*: No = number of participants; dmft = dental caries in deciduous dentition

Values are percentages for categorical variables, means (SD) for continuous variables with a normal distribution, or medians (95% range) for continuous variables with a skewed distribution; Differences were tested using one way ANOVA and Chi‐squared tests for variables with normal distribution and Kruskal‐Wallis Nonparametric test for, using Europeans as the reference group; Significant *p*‐values are presented in italic font.

a Dental age calculated by the Dutch standard.

b Dental age calculated by the French‐Canadian standard.

c Dental age calculated by the International Demirjian standard.

**Table 3:** The association between ancestry and dental development (dental age)

	Model 1			Model 2		
	β	95%CI	*p*‐value	β	95%CI	*p*‐value
*a. Geographic ancestry*						
Dutch (reference)	–	–	–	–	–	–
Cape Verdean	0.11	−0.03, 0.24	0.122	0.01	−0.12, 0.15	0.845
Moroccan	0.20	0.09, 0.30	*<0.001*	0.18	0.07, 0.28	*0.001*
Turkish	0.27	0.18, 0.37	*<0.001*	0.22	0.12, 0.32	*<0.001*
Dutch Antillean	0.35	0.21, 0.50	*<0.001*	0.27	0.12, 0.41	*<0.001*
Surinamese Creole	0.24	0.10. 0.38	*0.001*	0.16	0.03, 0.30	*0.020*
Surinamese Hindustani	0.10	−0.04, 0.24	0.155	0.10	−0.03, 0.24	0.137
*b. Genetic ancestry*						
1. Total (*N* = 2,786)						
European content of ancestry	−0.37	−0.49, −0.25	*<0.001*	−0.32	−0.44, −0.20	*<0.001*
African content of ancestry	0.41	0.27, 0.55	*<0.001*	0.29	0.16, 0.43	*0.001*
Asian content of ancestry	0.19	−0.01, 0.39	0.066	0.28	0.09, 0.48	*0.005*
2. Europeans (*N* = 2,473)						
European content of ancestry	−0.69	−0.93, −0.45	*<0.001*	−0.63	−0.87, −0.40	*<0.001*
African content of ancestry	0.68	0.38, 0.99	*<0.001*	0.57	0.27, 0.87	*<0.001*
Asian content of ancestry	0.64	0.27, 1.01	*0.001*	0.62	0.26, 0.98	*0.001*

*Abbreviations*: β = regression coefficients; CI = confidence interval; genetic contents of ancestry are investigated as continuous variables; Significant *p*‐values are presented in italic font.

The dental age calculated by the Dutch standard was higher in children of African ancestry (mean: 10.65 years) compared with children of European ancestry (mean: 10.32 years). Dental age in children of Asian ancestry (mean: 10.31 years) was not significantly different (*p* = 0.900) compared with children of European ancestry.

The nonresponse analysis showed that children who did not participate in the follow‐up measurements of dental development differed significantly in age, height, and dmft from those with follow‐up measurements (Supporting Information Table S1).

### 3.2 The association between geographic ancestry and dental age

In Model 1, Moroccan (β = 0.20; 95% CI: 0.09, 0.30), Turkish (β = 0.27; 95% CI: 0.18, 0.37), Dutch Antillean (β = 0.35; 95% CI: 0.21, 0.50), and Surinamese Creole (β = 0.24; 95% CI: 0.10, 0.38) children preceded Dutch children in dental development (Table 3a). No differences in dental age were found either between Cape Verdean and Dutch children (β = 0.11; 95% CI: −0.03, 0.24), or between Surinamese Hindustani and Dutch children (β = 0.10; 95% CI: −0.04, 0.24). After adjusting for hypodontia, BMI, height, and dmft (Model 2) the association remained significant, however the effect estimates decreased from 10% to 40% (Moroccan [β = 0.18; 95% CI: 0.07, 0.28], Turkish [β = 0.22; 95% CI: 0.12, 0.32], Dutch Antillean [β = 0.27; 95% CI: 0.12, 0.41], and Surinamese Creole [β = 0.16; 95% CI: 0.03, 0.30] children preceded Dutch children in dental development). Again, no difference on dental age was found either between Cape Verdean and Dutch children (β = 0.01; 95% CI: −0.12, 0.15), or between Surinamese Hindustani and Dutch children (β = 0.10; 95% CI: −0.03, 0.24).

### 3.3 The association between the genetic content of ancestry and dental age

#### 3.3.1 Total population

In Model 1, the increase in European ancestral content was associated with lower dental age (β = −0.37; 95% CI: −0.49, −0.25) (Table 3b.1). After adjusting for hypodontia, BMI, height, and dmft (Model 2) the association remained, however the effect estimate was attenuated (β = −0.32; 95% CI: −0.44, −0.20). In contrast, the increase in African ancestral content was associated with higher dental age (β = 0.41; 95% CI: 0.27, 0.55) in Model 1. After adjusting for hypodontia, BMI, height, and dmft (Model 2) the effect estimate decreased (β = 0.29; 95% CI: 0.16, 0.43). No statistically significant association was revealed between Asian ancestral content and dental age in Model 1(β = 0.19; 95% CI: −0.01, 0.39) which is only adjusted for chronological age. In contrast, after additionally adjusting for hypodontia, BMI, height, and dmft in Model 2, the increase in Asian ancestral content was statistically significantly associated with higher dental age (β = 0.28; 95% CI: 0.09, 0.48).

#### 3.3.2 European children

When the above analysis was performed in European children only (their fraction of estimated European ancestry was higher than 50%), who represented the majority of our study population and a more homogeneous sample, the associations remained in the same directions for each genetic ancestral content (Table 3b.2). Considering all the potential confounders, Model 2 revealed a significant association of European ancestral content with delayed dental age (β = −0.63; 95% CI: −0.87, −0.40). In contrast, the African ancestral content (β = 0.57; 95% CI: 0.27, 0.87) and Asian content of ancestry (β = 0.62; 95% CI: 0.26, 0.98) were both significantly associated with an advanced dental age in European children.

### 3.4 The association between genetic ancestry and development of each left mandibular tooth

Taking potential confounders into consideration, Model 2 revealed significantly higher developmental stages for the canine (β = 0.40; 95% CI: 0.10, 0.69), first premolar (β = 0.42; 95% CI: 0.14, 0.70), second premolar (β = 0.48; 95% CI: 0.20, 0.76), and first molar (β = 1.62; 95% CI: 0.21, 3.03) in children of African ancestry compared to children of European ancestry (Supporting Information Figure S2). Both Model 1 and Model 2 did not reveal any significant difference in developmental stages of each left mandibular tooth in children of Asian ancestry compared with children of European ancestry (Supporting Information Figure S3). As the central and lateral incisors were in the final stages of development, ordinal regression analyses were not preformed because of the lack of sufficient variability.

## 4 DISCUSSION

In this multi‐ethnic, population‐based prospective cohort study of 10 year‐old children born in the Netherlands, those of Moroccan, Turkish, Dutch Antillean, and Surinamese‐Creole descent showed a 2‐to‐4 month advanced dental development compared to those of Dutch descent. Cape Verdean and Surinamese Hindustani children did not significantly differ in dental development compared with Dutch children. Further, the increase in European ancestral content was associated with a deceleration in dental development of approximately 4‐to‐5 months. In contrast, the increase in African ancestral content was associated with an acceleration in dental development of approximately 3‐to‐5 months, and the increase in Asian ancestral content was associated with an acceleration in dental development of approximately 3 months. The effect estimates of the European, African and Asian ancestral contents in dental development doubled when investigated only in the European children.

**Figure 1:**
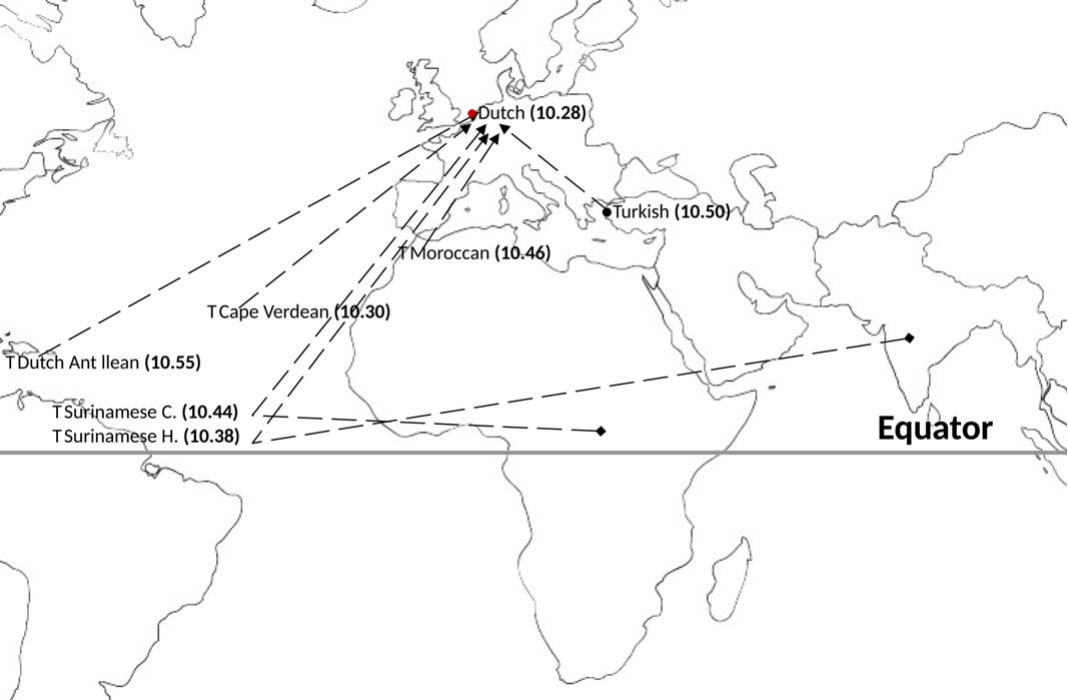
Schematic presentation of dental age for each geographic ancestry. The numbers in brackets and bold font represent the LS (least square) mean of dental age for each ethnic group, adjusted for age, hypodontia, BMI, height, and dmft; The lines in dashes show the migration of each ethnic group from the place of origin to the Netherlands; Surinamese C. (Creole) and Surinamese H. (Hindustani)

**Figure 2:**
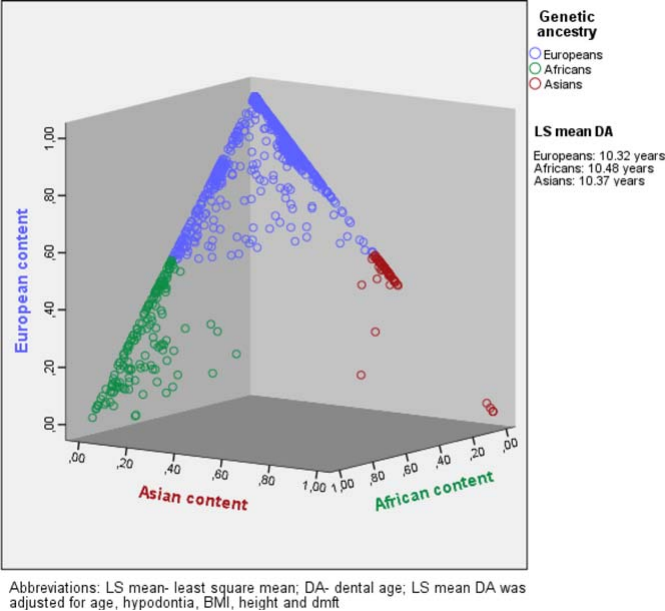
Graphic presentation of dental age for each genetic ancestry based on proportions (%) of European, African, and Asian ancestral content. LS mean—least square mean; DA—dental age; LS mean DA was adjusted for age, hypodontia, BMI, height, and dmft Addition: The highest reached fraction of estimated ancestry proportions such as European content, African content and Asian content (presented as *x*, *y*, and *z* axes in sides of cub) assigned children to one of the three ancestry groups Europeans, Africans, or Asians

The results of the current study are consistent with the seminal work from Garn and Roberts (Garn, Lewis, & Blizzard, 1965; Garn, Lewis, & Kerewsky, 1965; Garn, Nagy, Sandusky, & Trowbridge, 1973; Garn & Russell, 1971; Roberts, 1969). Garn and colleagues explored the influence of genetic, nutritional, and economic factors on variation in human dental development. Considering also the findings of our study, genetic ancestral content is an important indicator for the acceleration of dental development. However, factors related to the environment, such as physical factors (sun exposure, temperature, humidity, altitude), cultural habits in nutrition, and hormonal levels, could be important determinants affecting dental development and modulating effects of genetic ancestry (Bogin, 1999; Roberts, 1978). According to the geographical context, Dutch Antillean revealed the highest dental age (Figure 1). According to the genetic perspective, this ethnic group also reaches high proportion in African ancestral content. As African children had the highest dental age (Figure 2), there is consistency in findings from both a geographic and a genetic perspective.

The acceleration of dental maturity is recognized as an indicator of pubertal growth spurts (Chertkow, 1980). Based on the geographic ancestry in this study, Dutch Antillean children, followed by Turkish, Moroccan, and Surinamese Creole children, were the most advanced in dental development. Previous studies in the Netherlands have shown that children of Turkish and Moroccan descent start puberty later than Dutch children, however they pass through the pubertal stages faster than the Dutch children (Fredriks et al., 2003; Fredriks et al., 2004). Lacking information on sexual maturity and given the young age of our sample, the association between the timing of dental development and puberty will be of high priority in future research in our cohort when children will be approximately 13 years old. Referring to the current literature, puberty occurs earlier in children of African descent compared to children of European descent (Lum et al., 2015). Taken into context, the completion of root formation of the mandibular canine (Stage “7”of development) and prior to apical closure (Stage “8”of development) may serve as a clinically useful indicator of pubertal growth spurts (Chertkow, 1980). In our study, African children exceeded European children in the development of the mandibular canine, first premolar, second premolar, and first molar (0.4–1.6 stages). Whether acceleration in the development of these teeth might be associated with any initial sign of puberty remains a matter of future investigations.

Genetic studies confirm that the majority of the variations exist within a population made of different ethnic groups rather than between large populations (Jorde et al., 2000; Latter, 1980). Accordingly, recent studies have demonstrated variations of dental maturity within a population (Liversidge et al., 2006; Liversidge et al., 1999; Nystrom et al., 1988). The strength of our study is the inclusion of a large number of subjects from a multi‐ethnic population‐based prospective cohort design, with ascertained measurements of dental development. Based on the colonial and working immigration history, the largest ethnic minority groups in the Netherlands are Cape Verdean, Dutch Antillean, Moroccan, Surinamese‐Creole, Surinamese‐Hindustani, and Turkish (Netherlands, 2003). Both geographic and genetic transition may play an important role for the differences in dental development (Townsend, Bockmann, Hughes, & Brook, 2012; Townsend & Brook, 2008). Thus, specifying the ancestry based on geography and genetics in our study adds more insight to the understanding of dental maturity in populations with heterogeneous ethnic backgrounds. The geography context distinguished more ethnicities, and differences in dental development were investigated between more geographic ancestral groupings, consequently (Figure 1). However, apart from the reference group of children, the other ethnic groups were of relatively small sample size. Furthermore, as all children were born in the Netherlands, there is added difficulty in accurately distinguishing between the ethnic groups. We did not distinguish between the first‐ and second‐generation migrants, and also did not take into account the existence of heterogeneity within ethnic groups, which may have attenuated our results. Therefore, we also used the genetic ancestry in the present study as an objective approach. One limitation of utilizing genetic ancestry is the simple categorization of the study population into distinct ancestral groupings, when no precise boundaries are recognized among populations (Bolnick, 2008). As the members of each of the groups classified as European, African, or Asian in this study are highly variable, the genetic analysis might not accurately separate genetic groups. Thus, in our main analysis, we considered genetic ancestry continuously based on European, African, and Asian genetic content for each individual. Furthermore, cases that did not reach any significant proportion of the three ancestral contents were excluded from the analysis. Another limitation to be counted is the small sample size of Asian children present in our study population, which might have affected the nonsignificant difference in developmental stages of each left mandibular tooth between European and Asian children. To decrease the heterogeneity related to the environmental component between Europeans, Africans and Asians when the study population is investigated as a whole, we further studied the influence of each genetic content of ancestry only in the European children. In this restricted and more homogenous sample, results held fairly consistently suggesting that the genetic ancestral content influences dental development.

A combination of several methods for determining dental development is generally recommended for a better estimation of dental age (Ben‐Bassat, Babadzhanov, Brin, Hazan‐Molina, & Aizenbud, 2014). We used three different dental age standards (Dutch, French‐Canadian, and International Demirjian standard) in order to obtain the best approximation of dental age. The three standards converged at roughly the same dental age for a given child, and the concordance of the three polynomial functions to the study population resulted to be low to moderate (*R* ^2^ = 0.06–0.32), consequently. Longitudinal measurements of dental development would be necessary to definitively prescribe the dental age standard that would best represent dental development of our study population. The Demirjian method assessing dental development is the most applicable method worldwide, making possible comparisons of findings obtained across different populations. Few studies in Europe have previously investigated ethnic differences in dental development, applying Demirjian's method. Nystrom et al. reported that northeastern Finnish children precede southeastern Finnish children in dental development, suggesting that differences in dental development within a homogeneous population should be considered when using the national charts (Nystrom et al., 1988). One decade later, Liversidge et al. reported no difference in dental development between British children of white Caucasian origin and British children of Bangladeshi origin; a nonsurprising finding for the authors because of the similar physiological growth of children with these origins (Liversidge et al., 1999). Subsequently, Liversidge et al. reported no difference in stages of development among children coming from eight different countries (Liversidge et al., 2006). In contrast, our findings showed differences in timing of dental development within a multi‐ethnic population, adding to the current literature that differences in dental development need to be considered in populations with heterogeneous origin when using the national charts.

Despite all regression models in the current study being adjusted for potential confounders, such as hypodontia, BMI, height, and dmft, residual confounding remains and important consideration. The effect of hypodontia, BMI, and height on dental development stood out in all analyses as being significant predictors of dental development (*p* < 0.001). Hypodontia showed a negative effect on dental development, whereas the BMI and height showed a positive effect on dental development within our population. The findings of this study were in accordance with the existing literature, as hypodontia is recognized as an indicator of delayed dental development. Conversely, BMI and height are recognized as indicators of advanced dental development (Filipsson & Hall, 1975; Hedayati & Khalafinejad, 2014; Tunc, Bayrak, & Koyuturk, 2011; Uslenghi, Liversidge, & Wong, 2006). In our investigation, BMI and height explained at the maximum 13% of the variation in dental development between ancestral groups. The small value of explained variance from BMI and height can be attributed to the fact that dental development is predominately under genetic control, with a less‐prominent role of environmental factors such as nutrition. BMI and height may simply explain more about the physiological growth in children, and thus ancestral differences in the general growth and development of children needs to be further explored to determine the extent of unique and overlapping components with dental development. Lastly, selection bias cannot be excluded as it is difficult to assess whether the associations of geographic and genetic ancestry with dental development of children were different between those included and those not included in the final study sample. However, many of the characteristics of the current study were highly representative of the catchment area of Rotterdam.

In conclusion, based on a geographic and genetic perspective, differences in dental development exist in a heterogeneous population with regard to the ancestral background. The approach of this study is appropriate for orthodontists to detect whether dental development of a child happens “faster” or “slower” at a fixed age in comparison with children of the same age but of a different ethnicity.

## Supporting information

Additional Supporting Information may be found online in the supporting information tab for this article.

Supporting Information 1
