# Supplementary material for: Ancestry and dental development: A geographic and genetic perspective
Source: Am J Phys Anthropol. 2017 Nov 15;165(2):299–308. doi: 10.1002/ajpa.23351 (PMC5813218; doi:10.1002/ajpa.23351)
Supplement: Supplementary file 1 — Supporting Information 1 [file AJPA-165-299-s001.docx]

**Supplementary Material**

**Ancestry and dental development: A geographic and genetic perspective**

**Brunilda Dhamo^1,2^, Lea Kragt ^1,2^, Olja Grgic ^1,2, 4^ , Strahinja Vucic ^1,2^, Carolina Medina-Gomez ^2,4^ , Fernando Rivadeneira ^2,3,4^ , Vincent WV Jaddoe^2,3^, Eppo B Wolvius^1,2^, Edwin M Ongkosuwito ^1,2*^**

**^1^**Department of Oral & Maxillofacial Surgery, Special Dental Care and Orthodontics, Erasmus University Medical Centre, Rotterdam, the Netherlands ; **^2^**The Generation R Study Group, Erasmus University Medical Centre, the Netherlands; ^3^ Department of Epidemiology, Erasmus University Medical Centre, the Netherlands; ^4^ Department of Internal Medicine, Erasmus University Medical Center Rotterdam, the Netherlands

***Corresponding author**: Edwin M Ongkosuwito

Department of Oral & Maxillofacial Surgery, Special Dental Care and Orthodontics,

Erasmus University Medical Centre

PO Box 2040, 3000 CA Rotterdam, the Netherlands

Tel +31 10 7036426

**Email**: e.ongkosuwito@erasmusmc.nl

**Short Title**: The influence of ancestry on dental development

**Figure S1. Flowchart of the participants included in the study**

Children participating in the Generation R Study **N=9,901**

**N=4,099** due to loss of follow-up at 9 year visit or withdrawal of consent

Children available for follow up measurements at the age of 9 year **N=5,862**

**N=156** excluded due to twin births

Singleton births available for follow up measurements at the age of 9 year **N= 5,706**

**N=2,096** excluded due to lack of accurate information on genetic content of ancestry contents

**No=1,091** excluded due to lack of accurate information on geographic ancestry

Singleton births with available information on geographic ancestry **N=4,615**

Children with available information on genetic content of ancestry **N=3,610**

**N=1,015** excluded due to no available DPR present

**N=824** excluded due to no available DPR present

Children specified as European, African or Asian based on their genetic content of ancestry **N=2,786**

Children (singleton births) with available measurements on geographic ancestry and dental development **N=3,600**

| **Table S1.** General characteristics of the non-participants in the follow-up measurements of dental development | | | | | | |
| --- | --- | --- | --- | --- | --- | --- |
| **Available information** | **Geographic ancestry** | | | **Genetic ancestry** | | |
|  | **Participation** (N=3600) | **No-Participation** (N=1015) | **p-value** | **Participation** (N=2786) | **No-Participation** (N=824) | **p-value** |
| **Age** | 9.81 (0.35) | 9.76 (0.46) | *<0.001* | 9.82 (0.34) | 9.77 (0.48) | *0.001* |
| **Sex** |  |  | 0.104 |  |  | 0.098 |
| Boys | 1810 (50.3) | 487 (48.0) |  | 1387 (49.8) | 388 (47.1) |  |
| Girls | 1790 (49.7) | 528 (52.0) |  | 1399 (50.2) | 435 (52.8) |  |
| Missing | - | - | - | - | 1 (0.0) |  |
| **Maternal age** | 31.04 (4.87) | 30.70 (5.09) | 0.054 | 30.91 (4.81) | 30.95 (4.88) | 0.839 |
| Missing |  |  |  |  | 1(0.0) |  |
| **Height** | 141.77 (6.62) | 141.19 (6.42) | *0.018* | 141.87 (6.75) | 141.53 (6.22) | 0.222 |
| Missing |  |  |  |  | 107 (13.0) |  |
| **Weight** | 35.51 (7.36) | 35.09 (7.04) | 0.125 | 35.47 (7.17) | 35.25 (6.97) | 0.469 |
| Missing |  |  |  |  | 106 (12.9) |  |
| **BMI** | 17.56 (2.76) | 17.52 (2.77) | 0.670 | 17.52 (2.66) | 17.53 (2.78) | 0.961 |
| Missing |  |  |  |  | 107 (13.0) |  |
| **dmft** | 0.0 (0.0-6.0) | 0.0 (0.0-6.0) | *<0.001* | 0.0 (0.0-7.0) | 0.0 (0.0-7.1) | *<0.001* |
| Missing | 878 (24.4) | 276 (27.2) |  | 650 (23.3) | 227 (27.5) |  |
| *Abbreviations*: No- number of participants, dmft-dental caries in deciduous dentition; Values are percentages for categorical variables, means (SD) for continuous variables with a normal distribution, or medians (95% range) for continuous variables with a skewed distribution; Differences were tested using independent t-test for continuous variables, chi-squared test for categorical variables and Kruskal-Wallis Non-Parametric test for variables with a skewed distribution, using participation group as the reference; Significant p-values are presented in italic font | | | | | | |

**Figure S2.** The association between genetic ancestry (Africans vs Europeans) and the development of each left mandibular tooth

*Abbreviations*: Model 1 is adjusted for age and sex; Model 2 is additionally adjusted for hypodontia, BMI, height and dmft (dental caries in deciduous dentition); the statistically significant parameter estimates are presented inside the squares

**Figure S3.** The association between genetic ancestry (Asians vs Europeans) and the development of each left mandibular tooth

*Abbreviations*: Model 1 is adjusted for age and sex; Model 2 is additionally adjusted for hypodontia, BMI, height and dmft (dental caries in deciduous dentition)
